# Peptide Cross-Linked Poly (Ethylene Glycol) Hydrogel Films as Biosensor Coatings for the Detection of Collagenase †

**DOI:** 10.3390/s19071677

**Published:** 2019-04-08

**Authors:** Norlaily Ahmad, Burcu Colak, De-Wen Zhang, Martin John Gibbs, Michael Watkinson, C. Remzi Becer, Julien E. Gautrot, Steffi Krause

**Affiliations:** 1 School of Engineering and Material Science, Queen Mary University of London, Mile End Road, London E1 4NS, UK; norlaily.ahmad@qmul.ac.uk (N.A.); b.colak@qmul.ac.uk (B.C.); m.j.gibbs@qmul.ac.uk (M.J.G.); j.gautrot@qmul.ac.uk (J.E.G.); 2 Centre of Foundation Studies, Universiti Teknologi MARA, Cawangan Selangor, Kampus Dengkil, Dengkil, Selangor 43800, Malaysia; 3 Institute of Medical Engineering, School of Basic Medical Sciences, Xi’an Jiaotong University Health Science Center, Xi’an 710061, China; zhangdewen@xjtu.edu.cn; 4 The Lennard-Jones Laboratories, School of Chemical and Physical Sciences, Keele University, Staffordshire ST5 5BG, UK; m.watkinson@keele.ac.uk; 5 Department of Chemistry, University of Warwick, Coventry CV4 7AL, UK; remzi.becer@warwick.ac.uk

**Keywords:** protease biosensor, collagenase, poly(ethylene glycol) norbornene, QCM, hydrogel degradation, peptide cross-links, click chemistry

## Abstract

Peptide cross-linked poly(ethylene glycol) hydrogel has been widely used for drug delivery and tissue engineering. However, the use of this material as a biosensor for the detection of collagenase has not been explored. Proteases play a key role in the pathology of diseases such as rheumatoid arthritis and osteoarthritis. The detection of this class of enzyme using the degradable hydrogel film format is promising as a point-of-care device for disease monitoring. In this study, a protease biosensor was developed based on the degradation of a peptide cross-linked poly(ethylene glycol) hydrogel film and demonstrated for the detection of collagenase. The hydrogel was deposited on gold-coated quartz crystals, and their degradation in the presence of collagenase was monitored using a quartz crystal microbalance (QCM). The biosensor was shown to respond to concentrations between 2 and 2000 nM in less than 10 min with a lower detection limit of 2 nM.

## 1. Introduction

Proteases have been reported as biomarkers in many chronic diseases, such as cancer, cardiometabolic disease and lung disease [1,2,3]. Matrix metalloproteinases (MMPs) are among the most extensively studied proteases related to the pathology of diseases. One example is collagenase, which is classified as matrix metalloproteinase-1 (MMP-1) and was previously reported to be at elevated levels in synovial fluid and blood serum of rheumatoid arthritis (RA) (30 ± 2.9 nM_MMP-1 in synovial fluid [4]) and osteoarthritis (OS) patients (8.9 ± 1.7 nM MMP-1 in synovial fluid [4]), and correlated with inflammation in acute RA and chronic pathology of OS [4,5,6]. In RA and OS, the matrix of articular cartilage was degraded by the proteases. Collagenases were reported to cause the first degradation in triple-helical collagen in articular cartilage, before further degradation by other proteases [7]. The development of biosensors for the detection of proteases is important as point-of-care devices and also to help the further investigation of different diseases in clinical studies.

Disposable biosensors based on the degradation of hydrogel films have previously been introduced for the detection of the activity of various proteases [8,9,10,11,12,13]. The degradation of peptide cross-linked oxidised dextran hydrogel films was successfully monitored using quartz crystal microbalance (QCM) and impedance measurements at interdigitated electrodes in the presence of periodontal disease biomarkers human neutrophil elastase (HNE), cathepsin G and matrix metalloproteinase-8 (MMP-8) [8,9,12], and a non-specific biomarker for inflammation in multiple sclerosis MMP-9 [8]. A peptide cross-linked polyacrylamide hydrogel was used to detect HNE with a QCM, although the device showed relatively low sensor responses [13]. Different peptide cross-linkers were used in these studies, and the results showed high specificity of the biosensors to the enzymes of interest. However, the QCM response to proteases using this material displayed some problems, including delays in the change of the sensor signal and a lack of stability in the absence of the enzymes. The use of synthetic polymers in this application would have the advantage that their properties could more easily be tailored than those of a natural polymer such as dextran. Peptide cross-linked poly(ethylene glycol) hydrogel is a promising biomaterial that has been used widely for drug delivery and tissue engineering [14,15,16,17,18], albeit not for sensor applications. Poly(ethylene glycol) (PEG) is a hydrophilic polymer and has been reported to have anti-fouling properties [19]. The use of the dendritic architecture of PEG as a biosensor material is promising as it has more active sites for cross-linking. The cross-linking of four-arm PEG norbornene (PEGNB) with specific peptide sequences is likely to offer good sensor responses and high specificity based on the peptide cross-linker used. Previously, thiol-ene click chemistry was used for cross-linking between peptide and PEGNB for cell encapsulation to mimic extracellular matrix [20,21,22]. Thiol-ene click chemistry was reported to be highly effective at low macromolecule concentrations and under mild reaction conditions [23].

In this study, novel sensor materials for the detection of proteases were developed by depositing thin films of PEGNB cross-linked with a peptide using the thiol-ene reaction, and sensor responses were monitored using a QCM (also see [24]). The performance of the sensor materials was assessed with the detection of collagenase as a proof-of-concept system. 

## 2. Materials and Methods

### 2.1. Materials

Peptide GCRDVPMS↓MRGGDRCG (VPM) with a molecular mass of 1696.97 Da was purchased from Proteogenix, France. Collagenase Activity Colorimetric Assay Kit, collagenase from *Clostridium histolyticum* for general use Type I (Product number C0130, Lot SLBW6959, MW 125,000 Da), 4-arm poly(ethylene glycol) norbornene (MW 20,000 Da), tricine (*N*-[tris(hydroxymethyl)methyl]glycine, 99%), sodium chloride (99.5%), calcium chloride dihydrate (99%), phosphate-buffered saline (PBS) tablet, anhydrous toluene (99.8%), 3-(trimethoxysilyl)propyl methacrylate (98%) and trichloro(octadecyl)silane (90%) were purchased from Sigma. Concentrated sulfuric acid (95–98%), zinc chloride and hydrogen peroxide (30% H_2_O_2_ (*w*/*w*)) were purchased from Fluka (Bucharest, Rumania). Sodium hydroxide (1 M) was purchased from Fisher Chemicals, (Loughborough, UK). Microscopic glass slides (Menzel-glaser, 75 mm × 25 mm) from Thermo Scientific (Loughborough, UK) were used in sensor fabrication. Round glass cover slips (20 mm) were purchased from VWR (Lutterworth, UK). Polished, gold-coated QCM crystals (AT-cut, 10 MHz) were purchased from International Crystal Manufacturing Company, Inc. (Oklahoma City, OK, USA). All solutions were prepared with Milli-Q water (resistivity 18.2 MΩ·cm).

### 2.2. Collagenase Activity Measurement

The collagenase activity in PBS (pH 7.4) was measured based on the rate of degradation of synthetic peptide (FALGPA) using the Collagenase Activity Colorimetric Assay Kit. The absorbance was measured in a kinetic mode using a SPECTROstar Nano microplate reader (BMG LABTECH (Aylesbury, UK)) set at λ = 345 nm, 25 °C and 9.5 min. The collagenase activity calculated for 20 nM (12.5 µg/mL) was 9.23 µmol/min (9.23 U/mL, 1 U of collagenase hydrolysed 1.00 µmol FALGPA per min).

### 2.3. Peptide Degradation

Prior to the preparation of hydrogel films, the peptide cross-linker VPM was first shown to be degraded by collagenase. To confirm degradation of the VPM peptide by collagenase, 100 µM of VPM in PBS at pH 7.4 was incubated with 25 nM collagenase for 3 h at 37 °C. The sample before and after degradation was measured using a matrix-assisted laser desorption/ionisation-time of flight mass spectrometer (MALDI-ToF MS).

### 2.4. Sensor Fabrication

#### 2.4.1. Cleaning of QCM crystals

The gold-coated QCM crystals were cleaned with piranha solution (a mixture of 3:1, *v*/*v*, concentrated sulfuric acid and 30 wt% hydrogen peroxide) for 3 min and then rinsed thoroughly with Milli-Q water three times. The crystals were then blow-dried using nitrogen prior to use.

#### 2.4.2. Functionalized Glass Slide

Microscopic glass slides were first cleaned using piranha solution for 10 min. The glass slides were rinsed thoroughly using Milli-Q water and dried in an oven at 100 °C for 10 min. The clean glass slides were immersed in a beaker of 0.02% *v*/*v* of trichloro(octadecyl)silane in anhydrous toluene for 90 min and closed tightly with Parafilm to avoid moisture absorption. The modified glass slides were then rinsed with toluene, acetone and lastly with Milli-Q water before blowing dry using nitrogen.

#### 2.4.3. Mixture of Peptide and PEGNB for Hydrogels

Prior to the sensor fabrication, three stock solutions of PEGNB (20 kDa) and VPM peptide in PBS pH 6 were prepared at concentrations of 192.4 mg/mL and 190.9 mg/mL respectively. A hydrogel solution of 50% VPM cross-linked PEGNB was prepared by mixing 104.2 µL (20 mM, 100.18 mg/mL) of PEGNB stock solution with 17.8 µL (10 mM, 8.5 mg/mL) of VPM peptide stock solution and adding PBS pH 6 until a 200 µL total volume was reached. Lastly, 5 mol% (with respect to thiol) of the photo-initiator Irgacure 2959 in methanol was added and the mixture was kept away from sunlight and used while fresh. To obtain different degrees of cross-linking, 52.15 µL (15 mM, 12.75 mg/mL) or 69.5 µL (20 mM, 12.75 mg/mL) of VPM peptide was added to the same amount of PEGNB (104.2 µL, 20 mM, 100.18 mg/mL) to achieve 75% and 100% cross-links, respectively.

#### 2.4.4. Deposition of Hydrogel

The cross-linking of the hydrogel on the QCM crystal was performed through UV thiol-ene chemistry. The hydrogel mixture prepared in Section 2.4.3 was sandwiched between the clean gold-coated QCM and the functionalized glass slide prepared in Section 2.4.2 before UV-curing for 300 s (17 mW/cm^2^, 350–500 nm) using an Omnicure series 1500 UV lamp. The hydrogel-film coated QCM crystals were removed from the functionalized glass slide and stored in PBS buffer before use.

### 2.5. Rheological Characterisation of Hydrogels

The resulting mechanical properties of VPM cross-linked PEGNB hydrogel were characterised using photo-rheology (TA Discovery HR-3 hybrid rheometer) with a UV-activated setup. UV irradiation was started after 30 s and kept on for 300 s. The storage moduli of hydrogels with different degrees of cross-linking were measured during this time. The storage modulus indicates the stiffness of the hydrogel, which increased due to the formation of intermolecular crosslinks [25].

### 2.6. Monitoring of Hydrogel Film Degradation Using QCM

**Figure 1:**
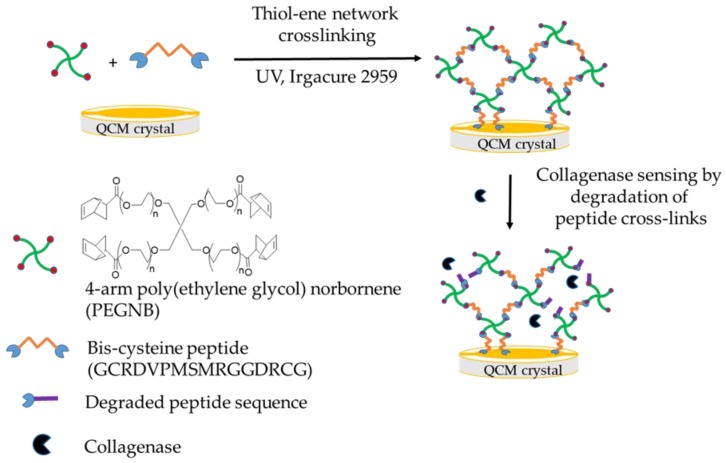
Schematic diagram of hydrogel formation and collagenase sensing. QCM: quartz crystal microbalance.

QCM measurements were conducted using an experimental setup that was modified from Sabot and Krause [11], and were carried out using a Hewlett-Packard HP 8751A (5 Hz–500 MHz) Network Analyser in reflectance mode. Quartz crystal admittance spectra were measured between the gold electrodes on the QCM crystals. One full quartz crystal admittance spectrum (402 points, acquisition time 1 s, AC stimulus 160 mV) was recorded over a range of 500 kHz centred at the QCM resonance frequency (~10 MHz) every 10 s. A set of sensors fabricated using VPM cross-linked PEGNB (20 kDa) were prepared for the detection of collagenase in the range of 0.2 to 2000 nM. The sensor was first equilibrated in PBS pH 7.4 to stabilize the signal, and the degradation was monitored over 10 min after the enzyme was added. The sensor response with different cross-link densities was also measured in the same QCM setup. The QCM admittance spectra were recorded before and after degradation. The QCM admittance spectra were then fitted with a Butterworth Van Dyke (BVD) equivalent circuit as described by Ballantine [26]. Two QCM parameters were used to describe the degradation of the hydrogel: the change in resistance, *ΔR*, and the change in reflective inductance, *ωΔL*. The change in resistance, *ΔR*, represents the viscoelasticity of the hydrogel or energy loss due to damping, and the change in reflective inductance, *ωΔL*, represents the mass loss of the hydrogel during the degradation. A schematic diagram of the hydrogel formation and collagenase sensing is shown in Figure 1.

## 3. Results and Discussion

### 3.1. Investigation of the Degradation of the VPM Peptide Sequence GCRDVPMSMRGGDRCG

**Figure 2:**
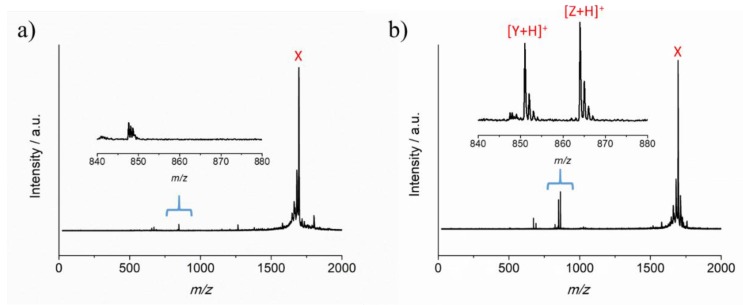
MALDI-ToF mass spectra of (**a**) VPM (GCRDVPMSMRGGDRCG) peptide, X; (**b**) Product of the degradation of X by collagenase.

As shown previously, peptide cross-linked hydrogels can be made specific for different target proteases by selecting appropriate peptide cross-linkers [9]. The peptide sequence GCRDVPMSMRGGDRCG (VPM) has been shown to be degraded by proteases such as matrix metalloproteinase-1 (MMP-1), matrix metalloproteinase-2 (MMP-2) and collagenase [27,28], and was therefore deemed to be a suitable model peptide cross-linker to assess the performance of the new sensor materials. For a specific clinical application, the peptide cross-linker would require optimization to achieve the required selectivity. To confirm that the VPM peptide sequence GCRDVPMSMRGGDRCG was degraded by collagenase, MALDI-ToF MS analysis was performed. Figure 2a shows the MALDI-ToF MS analysis of the monoisotopic mass of the peptide at *m*/*z* 1696, X, and was consistent with the structure expected of the native peptide GCRDVPMSMRGGDRCG. Three peaks were observed after degradation (Figure 2b), representing the non-degraded peptide sequence X as well as degraded sequences. The molecular ion at *m*/*z* 851.0, [Y+H]^+^ is consistent with the degraded sequence [MRGGDRCG+H]^+^ and the molecular ion at *m*/*z* 864.0, [Z+H]^+^ is consistent with the second degraded sequence [CGRDVPMS+H]^+^, confirming that collagenase degradation of the peptide sequence does occur.

### 3.2. Photo-Rheology of Hydrogel Films

**Figure 3:**
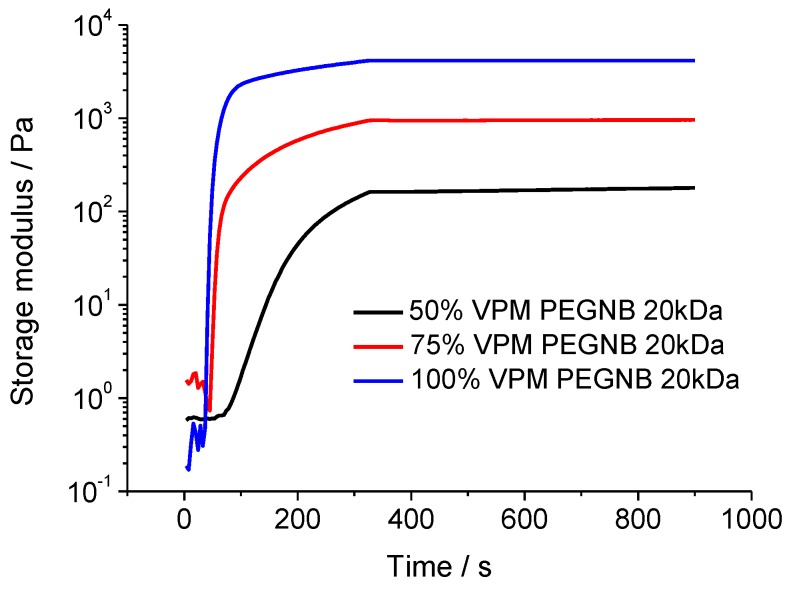
The evolution of the storage modulus as a function of time for different percent cross-linking of VPM with PEGNB (20 kDa).

As shown in Figure 3, photo-rheology confirmed the rapid evolution of the PEGNB-based hydrogel system as a function of time after UV exposure of the PEGNB–VPM peptide solutions with different peptide cross-linker concentrations. For the VPM cross-linked PEGNB (20 kDa) hydrogel (Figure 3), the storage modulus increased sharply for 75% and 100% cross-linking 30 s after the UV light was turned on, whilst the storage modulus for 50% cross-linking increased slowly. VPM cross-linked PEGNB (20 kDa) at 50%, 75% and 100% resulted in storage moduli of 198.6 ± 36.28 Pa, 968.5 ± 17.43 Pa and 4307 ± 377.9 Pa, respectively. As the initial peptide concentration in the hydrogel mixture increases, the cross-linking efficiency will increase and result in an increase of the storage modulus [21].

### 3.3. Optimisation of VPM Cross-Linked PEGNB Hydrogel with Different Degrees of Cross-Linking

**Figure 4:**
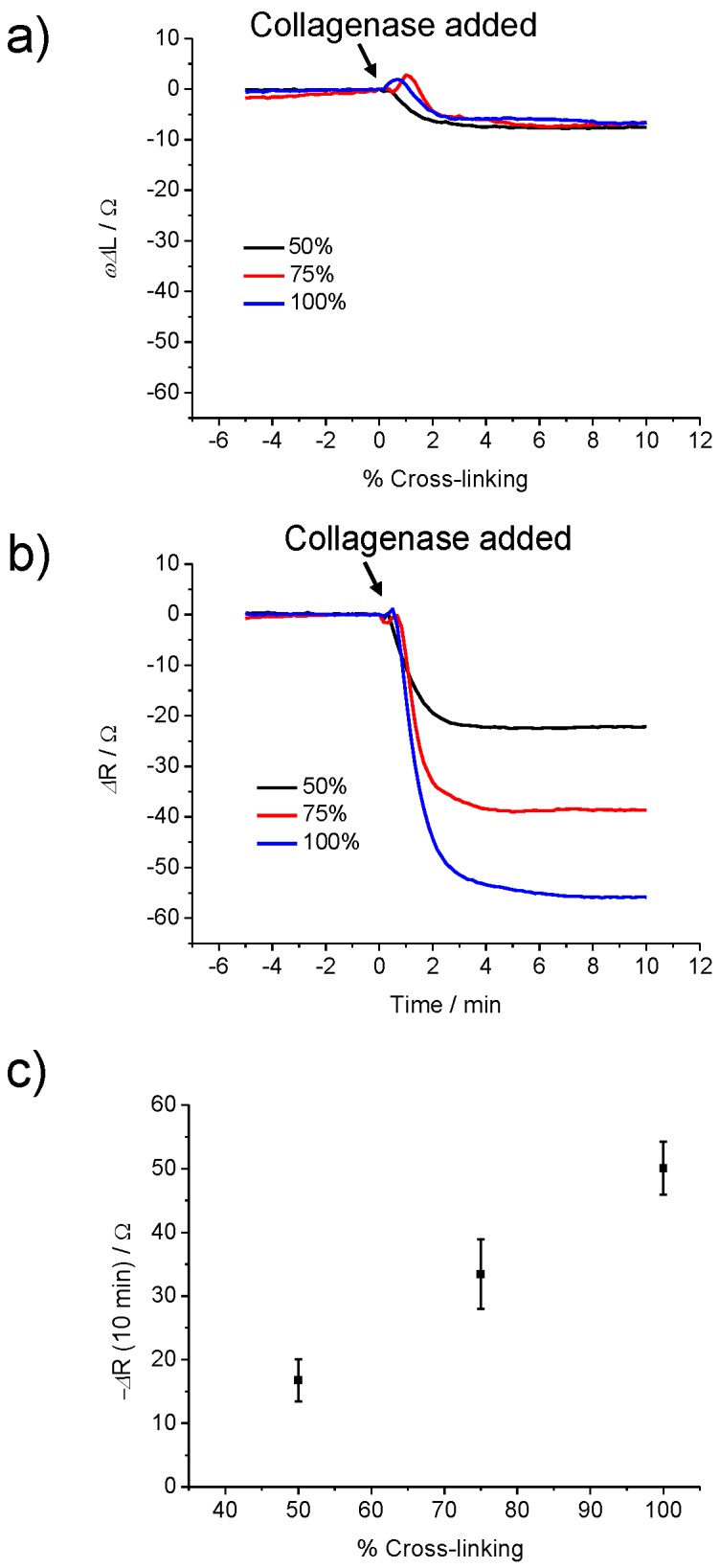
QCM response (**a**) *ωΔL* and (**b**) *ΔR* of 50%, 75% and 100% VPM cross-linked poly(ethylene glycol) norbornene (PEGNB; 20 kDa) before and after addition of 2000 nM collagenase at t = 0. (**c**) Average of *ΔR* for different degrees of cross-linking after 10 min incubation. The error bars represent the standard deviation for three individual sensors.

Prior to the degradation experiment, the hydrogel was first equilibrated in PBS pH 7.4 before exposure to collagenase and the degradation was recorded for 10 min after the enzyme was added (Figure 4). To optimise the effect of different degrees of cross-linking on the degradation of the hydrogel by collagenase, 50%, 75%, and 100% of VPM were used to cross-link with PEGNB (20 kDa) to form hydrogel films. The minimum amount of peptide cross-linker needed to form the cross-linked hydrogel with 4-armed PEGNB was 50%. As shown in Figure 4a,b, small increases in *ωΔL* and *ΔR* were observed immediately after the addition of 2000 nM collagenase, which corresponded to the enzyme binding to the hydrogel. Interestingly, for all degrees of cross-linking, *ωΔL* was observed to decrease by the same value after the enzyme was added (Figure 4a). The decrease in *ωΔL* was within the range of −6.5 to −7.6 Ω, which corresponds to a small mass loss from the hydrogel film. It is assumed that only a small portion of the hydrogel dissolved when the cross-links were cleaved by collagenase, as the hydrogel is covalently attached to the gold surface through the thiol groups in the terminal cysteine functionalities of the peptide. In contrast, the *ΔR* value during the degradation of the hydrogel films was observed to be dependent on the degree of cross-linking (Figure 4b). At 50%, 75% and 100% cross-linking, the average *ΔR* at 10 min observed were −16.7 Ω, −33.5 Ω and −50 Ω, respectively (Figure 4c). An increase of the cross-linking percentage caused an increase of the sensor response, due to the availability of more cleavage sites at higher degrees of cross-linking. The changes in the reactive inductance, *ωΔL*, were observed to be significantly lower than the changes of the resistance, *ΔR* (Figure 4a,b). However, there was no correlation of the mass loss with the cross-link density and, hence, the film stiffness (i.e., when cross-links were broken, there was some mass loss from the film due to the degradation, but this was counteracted by the binding of the enzyme to the peptide fragments in the films). The mass loss and the presence of the enzyme in the film made it stiffer, resulting in a decrease of *ΔR*. This binding most likely caused the deactivation of the enzyme as it could no longer diffuse and resulted in the incomplete degradation of the film.

### 3.4. Concentration-Dependent Collagenase Response of VPM Cross-Linked PEGNB Hydrogel

**Figure 5:**
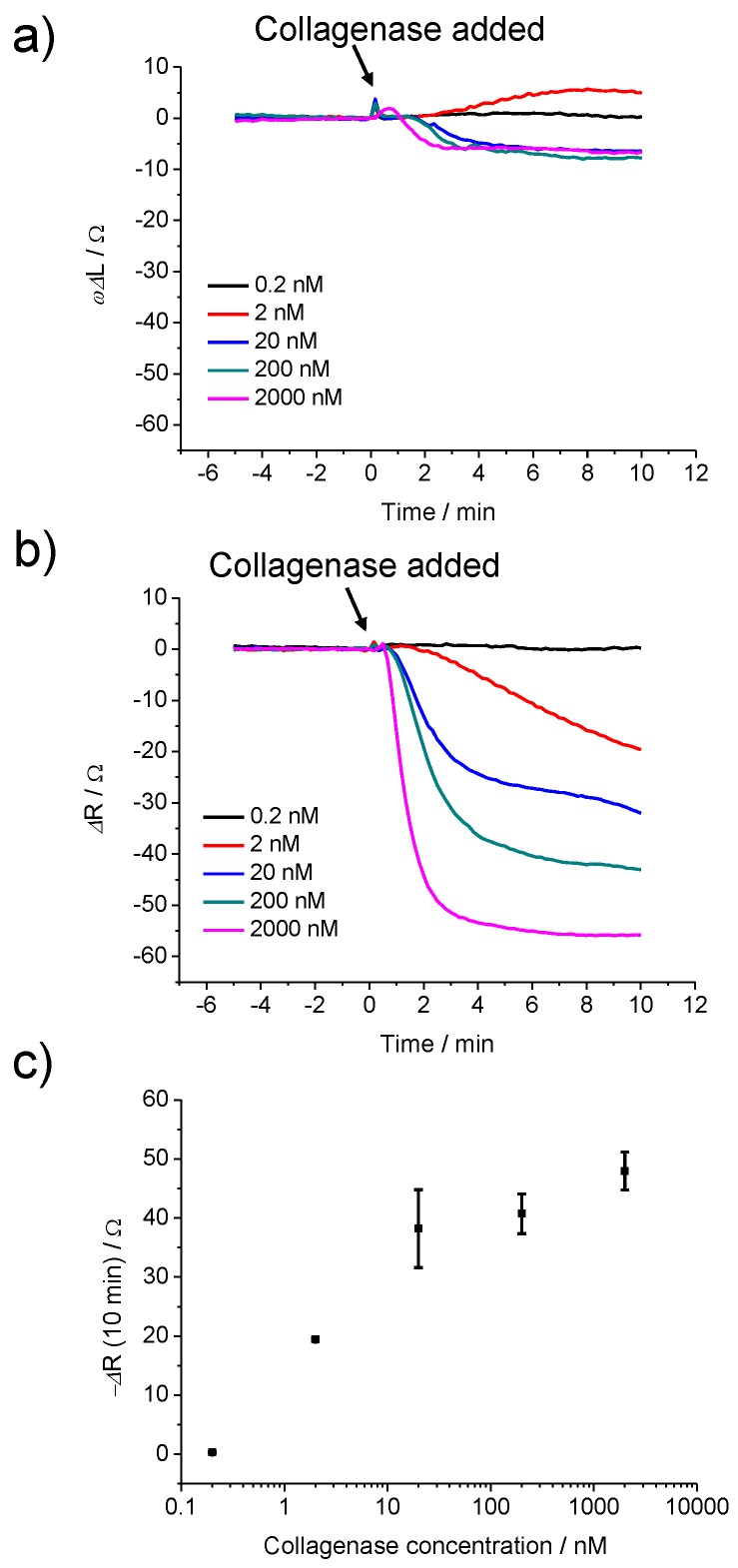
QCM response (**a**) *ωΔL* and (**b**) *ΔR* of 100% VPM cross-linked PEGNB (20 kDa) before and after exposure to different concentrations of collagenase. (**c**) Collagenase concentration dependence of the average of *ΔR* value based on the sensor response in panel (**b**). The error bars represent the standard deviation for three individual sensors.

The effects of different concentrations of collagenase on the optimised hydrogel, 100% VPM cross-linked PEGNB (20 kDa), were investigated as shown in Figure 5. The hydrogel was exposed to a range of 0.2 to 2000 nM of collagenase, and the degradation was monitored using QCM measurements. When the hydrogel was exposed to 0.2 nM of collagenase in PBS, no response in the changes of the reactive inductance, *ωΔL*, and the resistance, *ΔR*, was observed (Figure 5a,b). An unexpected but reproducible increase of *ωΔL* was observed when 2 nM collagenase was added. It is assumed that the mass increase that was caused by the enzyme binding to the peptide fragments outweighed the mass loss caused by the degradation. However, a small decrease in *ωΔL* was observed when the hydrogel was exposed to 20–2000 nM of collagenase. The rate of degradation increased with increasing collagenase concentration, but the total mass loss was similar for all concentrations >2 nM. An increase of the sensor response, *ΔR*, was observed as the concentration of collagenase increased (Figure 5b), which corresponds to a faster degradation of the peptide cross-links with the collagenase concentration. Not only the rate of change but also the total change of *ΔR* was concentration dependent, which indicates binding of the enzyme to the partially degraded film and the subsequent deactivation of the enzyme as discussed above.

## 4. Conclusions

A collagenase biosensor was successfully developed based on the degradation of peptide cross-linked PEGNB monitored by QCM measurements. The optimised sensor at 100% VPM cross-linking showed a concentration-dependent response within a range of 2 to 2000 nM collagenase. The lower detection limit of this sensor was 2 nM and the response was complete within 10 min. The relative inductance, *ωΔL,* in this system was very small and no significant differences were measured when testing with different degrees of cross-linking and different enzyme concentrations. The mass loss due to the degradation was most likely offset by binding of the enzyme to the partially degraded film. This binding caused a decrease in the resistance due to an increase in the stiffness of the film and also deactivated the enzyme, resulting in the incomplete degradation of the hydrogel.

Our findings suggest the efficacy of the new sensor materials for the detection of proteases. With further optimisation of the peptide sequence, we can expect improved selectivity and activity. Current investigations are ongoing in our laboratories along these lines, with a long-term aim of developing MMP-specific point-of-care sensors.
